# Visit-to-Visit Blood Pressure Variability and Cerebral White Matter Lesion Progression: A Pooled Individual Patient Data Analysis of 2 Trials

**DOI:** 10.1212/WNL.0000000000218302

**Published:** 2026-07-10

**Authors:** Wenbo Zhao, Yue Qiao, Zihan Sun, Eric L. Harshfield, Lupei Cai, Xunming Ji, Hugh S. Markus

**Affiliations:** 1 Department of Neurology, Xuanwu Hospital Capital Medical University, National Centre for Neurological Disorders, Beijing, China;; 2 Stroke Research Group, Department of Clinical Neurosciences, University of Cambridge, United Kingdom; and; 3 Beijing Institute for Brain Disorders, Capital Medical University, Beijing, China.

## Abstract

**Background and Objectives:**

Visit-to-visit blood pressure variability (BPV) may contribute to cerebral white matter lesions (WML) progression, but previous evidence has been inconsistent and limited by methodological heterogeneity. This study aimed to assess the association between BPV and WML progression and its mediating role in the neuroprotective effects of intensive blood pressure (BP) control.

**Methods:**

This post hoc individual participant data pooled analysis included the MRI substudies of ACCORD MIND and SPRINT MIND. Participants were eligible if they had both baseline and follow-up MRI scans and at least 3 BP measurements from the 3-month visit onward. Systolic BPV was calculated using multiple indices, with variation independent of mean (VIM) prespecified as the primary metric. WML progression was quantified as absolute and annualized changes in abnormal white matter (AWM) volume, with inverse hyperbolic sine (asinh)-transformed total change as the primary metric. Associations between systolic BPV and AWM volume changes were examined using multivariable linear regression, and causal mediation analysis assessed whether systolic BPV mediated the effects of intensive BP control.

**Results:**

A total of 952 participants (mean age 64.8 years [SD 7.1]; 400 [42%] women) were included, contributing a median of 12 (interquartile range 10–14) BP measurements. Median AWM volume increased from 1.69 mL to 2.58 mL (0.43 mL per year [SD 0.84]). Higher systolic BPV was independently associated with faster AWM volume progression (β = 0.017, 95% CI 0.001 to 0.033). In raw annualized terms, participants in the highest tertile of SBP-VIM had 0.160 mL/y faster AWM progression than those in the lowest tertile. Intensive BP control was associated with slower AWM volume progression (β = −0.270, 95% CI –0.393 to −0.146). Mediation analysis indicated that systolic BPV partially mediated the association between intensive BP control and reduced AWM volume progression (average causal mediation effect 0.014, 95% CI 0.001–0.034), accounting for 9.15% of the total effect.

**Discussion:**

Higher visit-to-visit systolic BPV was independently associated with faster WML progression. The benefit of intensive BP lowering on white matter integrity was partly mediated by reduced BPV, suggesting BP stability as a modifiable target to prevent white matter injury.

## Introduction

Cerebral small vessel disease (CSVD) is a highly prevalent cerebrovascular disorder causing both lacunar ischaemic stroke and intracerebral hemorrhage and is the most common pathology causing vascular cognitive impairment and dementia.^1,2^ On brain MRI, CSVD manifests as white matter lesions (WML), lacunes, enlarged perivascular spaces, and cerebral microbleeds.^3,4^ Among these imaging markers, WML, which strongly predict cognitive decline and loss of functional independence, are widely used as surrogate outcomes for disease progression.^5^

Hypertension is the most important modifiable risk factor for CSVD.^6^ Elevated blood pressure (BP) has been consistently associated with greater burden of WML across the full spectrum of BP values, even in individuals without clinically overt hypertension.^7,8^ Population-based studies further confirm that hypertension is one of the strongest determinants of WML burden, second to age.^9^ Longitudinal studies indicate that BP control can slow WML progression,^10^ and randomized trials have demonstrated that intensive systolic BP (SBP) lowering provides additional benefit. In the SPRINT MIND trial, intensive BP control significantly reduced WML progression compared with standard therapy,^11^ and similar effects were observed in the ACCORD MIND trial and a pooled meta-analysis.^12,13^ Despite these established benefits, the mechanisms underlying the protective effect of intensive BP control remain incompletely understood. Moreover, WML progression varies considerably among individuals with similarly well-controlled mean BP levels, suggesting that factors beyond mean BP may contribute to white matter injury.

Blood pressure variability (BPV), reflecting dynamic fluctuations in BP independent of mean levels, is increasingly recognized as a key determinant of CSVD burden and progression.^14-16^ In particular, higher BPV has been found to be associated with both the burden and progression of WML, independent of mean BP.^8,17,18^ However, most previous studies have been constrained by methodological limitations, including heterogeneous definitions of BPV, a limited number of BP measurements, and widely varying follow-up intervals ranging from days to years, which hinder comparability and reliability across studies. Moreover, it remains unclear whether BPV contributes to the neuroprotective effects of intensive BP control, and whether it accounts for the residual variability in WML progression among patients with well-controlled mean BP.

To address these questions, we conducted a post hoc individual participant data pooled analysis from 2 large randomized controlled trials (SPRINT MIND and ACCORD MIND). These trials evaluated intensive BP lowering in distinct populations and incorporated serial, standardized BP measurements, adjudicated MRI outcomes, and standardized follow-up intervals. Leveraging the rigorous design of these 2 trials, we aimed to (1) determine the independent association between visit-to-visit systolic BPV and WML progression and (2) evaluate whether BPV mediates the neuroprotective effects of intensive BP control on white matter integrity.

## Methods

### Study Design

We performed a pooled individual participant-level analysis of the Systolic Blood Pressure Intervention Trial Memory and Cognition in Decreased Hypertension (SPRINT MIND) and the Action to Control Cardiovascular Risk in Diabetes (ACCORD) Memory in Diabetes (MIND) trial. Both trials included MRI substudies, and datasets were obtained from the National Heart, Lung, and Blood Institute's Biologic Specimen and Data Repository Information Coordinating Center (BioLINCC^19^). Details of the trial designs, study populations, interventions, and study procedures have been published previously.^11,20^

The SPRINT MIND trial, a predefined substudy of SPRINT, investigated the effect of intensive SBP lowering to <120 mm Hg on the incidence of probable dementia and mild cognitive impairment compared with a target of <140 mm Hg. Brain MRI was performed in a subset of participants at baseline (n = 670) and at 4 years of follow-up (n = 449).^11^ The ACCORD MIND trial, a substudy of the ACCORD trial, assessed whether intensive compared with standard management of hyperglycemia, BP or lipid levels mitigated cognitive decline and brain atrophy in individuals with type 2 diabetes. In the ACCORD MIND trial, a subset of the participants underwent baseline MRI (n = 614) and follow-up MRI at 40 months (n = 503).^20^

### Standard Protocol Approvals, Registrations, and Patient Consents

The parent ACCORD and SPRINT trials were approved by the ethics committee at each participating site, and all participants provided written informed consent at enrollment in the parent trials. The present post hoc individual participant data pooled analysis used deidentified trial data, and, in accordance with institutional policy, additional ethics approval was not required for the present analysis (reference PRE.2025.052). The requirement for additional individual participant consent was waived. The parent trials were registered at ClinicalTrials.gov (ACCORD MIND: NCT00000620; SPRINT MIND: NCT01206062).

### Participants

To achieve a stable SBP, participants in the intensive BP control group typically required successive adjustments to their antihypertensive regimen during the first 3 months after enrollment.^21^ Therefore, to ensure reliable estimation of BPV, only BP measurements obtained from the 3-month visit onward were included in the calculation. Participants were eligible if they had both baseline and follow-up MRI assessments and at least 3 standardized BP measurements after the 3-month visit. Accordingly, participants were excluded if they had fewer than 3 BP measurements between the 3-month visit and the MRI follow-up (3–48 months in the SPRINT MIND trial and 3–40 months in the ACCORD MIND trial).

### Blood Pressure Measurements and Variability

In both the included trials, BP was measured at each clinic visit using validated automated devices (Omron 907XL or Omron 907). Participants were seated, and 3 consecutive readings were obtained, with the mean recorded as the visit BP. In SPRINT MIND trial, visits occurred monthly for the first 3 months and every 3 months thereafter. In ACCORD MIND trial, visit frequency varied by treatment assignment: participants in intensive glycemia or intensive BP groups were seen monthly for the first 4 months, then every 2 months; those in standard treatment groups were seen every 4 months (eFigure 1).

BPV was calculated using BP measurements obtained from 3 months after enrollment until the MRI follow-up, as antihypertensive regimens were often being adjusted during the initial period to achieve stable BP levels. Four metrics were calculated: (1) SD of visit-to-visit BP; (2) coefficient of variation (CV), calculated as SD divided by mean BP; (3) average real variability (ARV), defined as the mean absolute difference between consecutive BP measurements; and (4) variation independent of mean (VIM), calculated as SD divided by mean BP raised to a power *k*, with *k* estimated from the population-specific regression of log(SD) on log(mean BP). In this study, systolic BPV was assessed and VIM was chosen as the primary metric for evaluating associations with WML progression because of its mathematical independence from mean BP, whereas SD, CV, and ARV were included in sensitivity analyses to assess the robustness of the results.

### Outcomes and Measures

MRI acquisition and processing procedures for both trials have been described in detail previously.^11,20^ Briefly, in the ACCORD MIND trial, image analysis was performed based on an automated multispectral computer algorithm that classified supratentorial brain tissue into CSF, gray matter, or white matter. Gray and white matter were further classified as normal or abnormal, and total brain volume was calculated by summing all gray and white matter regions of interest. In the SPRINT MIND trial, a similarly automated pipeline was used to preprocess structural MRI scans, including correction of intensity inhomogeneity and multi-atlas skull stripping for intracranial tissue extraction. Anatomical regions of interest were identified using a multi-atlas label-fusion method and were used to segment supratentorial gray and white matter tissues, with total brain volume defined as the sum of segmented gray and white matter.

In the SPRINT MIND trial, the primary imaging endpoint was initially defined as the change in total CSVD lesion volume and was later refined to the change in WML volume. Data on both abnormal white matter (AWM) and WML were available. In contrast, the ACCORD MIND trial provided data only on AWM volume. AWM represents the volume of white matter voxels classified as abnormal by automated MRI segmentation. It consists predominantly of WML of presumed microvascular origin and encompasses both focal white matter hyperintensities and the surrounding regions of diffuse CSVD-related tissue injury. Thus, AWM provides a comprehensive volumetric measure of the burden of CSVD-related white matter abnormalities.^22,23^ Accordingly, AWM volume was selected as the imaging outcome to evaluate WML progression in this pooled analysis.

The primary outcome of this pooled analysis was progression of AWM volume, quantified as the total change from baseline to follow-up MRI (48 months in SPRINT MIND and 40 months in ACCORD MIND) and transformed using the inverse hyperbolic sine (asinh) function for the primary analysis. AWM volume was measured according to each trial's predefined segmentation and quality control protocols. Change in AWM volume was expressed as both absolute and annualized differences from baseline and served as the dependent variable in all analyses. The main analysis evaluated the association between visit-to-visit systolic BPV and AWM volume progression, independent of mean BP levels. Exploratory analyses further examined whether systolic BPV mediated the effect of intensive systolic BP lowering on AWM volume progression.

### Statistical Analyses

Continuous variables were summarized as mean (SD) or median (interquartile range [IQR]), and categorical variables as counts and percentages. Baseline characteristics were summarized by tertiles of SBP-VIM, and trends across tertiles were assessed by modeling SBP-VIM tertiles as an ordinal variable.

The associations between BPV and AWM volume progression were examined using multiple linear regression models, with β coefficients and corresponding 95% CIs. SBP-VIM was analyzed both as a continuous variable and as tertiles (T1, T2, T3). Change in AWM volume was operationalized in 4 ways: (1) the total change in asinh-transformed volume, (2) the total change in raw volume (mL), (3) the annualized change in asinh-transformed volume, and (4) the annualized change in raw volume (mL/y). The total change in asinh-transformed volume was prespecified as the primary metric. Three hierarchical models were fitted: model 1 adjusted for age, sex, and ethnicity (White, Black, Hispanic, or Other); model 2 additionally adjusted for mean SBP during the study period; and model 3 further adjusted for total brain volume, intensive BP control, hypertension, diabetes, history of stroke, smoking status, high-density lipoprotein cholesterol, and body mass index. Sensitivity analyses used alternative systolic BPV indices (SD, CV, and ARV). Restricted cubic spline models with 4 knots were used to explore a potential nonlinear association between systolic BPV and AWM volume progression, adjusted for model 3 covariates.

Subgroup analyses assessed whether the association between SBP-VIM and AWM volume progression varied across clinically relevant strata, including age (<60 and ≥60 years), sex, ethnicity, intensive BP control, smoking status (current smoker or not), history of cardiovascular disease, history of congestive heart failure, history of atrial fibrillation, diabetes, hypertension, and history of stroke. For each subgroup, fully adjusted β coefficients (95% CIs) were estimated from multivariable linear regression models (model 3). Multiplicative interaction terms were introduced to test for statistical heterogeneity across subgroups.

The association between intensive BP control and AWM volume progression was also examined using multiple linear regression models. Two models were fitted: model 1 adjusted for age, sex, and ethnicity (White, Black, Hispanic, or Other); model 2 additionally adjusted for total brain volume, hypertension, diabetes, history of stroke, smoking status, high-density lipoprotein cholesterol, and body mass index.

To assess whether the effect of intensive BP control on AWM volume progression was mediated through systolic BPV, we performed causal mediation analysis using nonparametric bootstrapping with 5,000 simulations to estimate bias-corrected 95% CIs for the average causal mediation effect (ACME), average direct effect (ADE), total effect, and proportion mediated. All mediation models were adjusted for key covariates (age, sex, ethnicity, total brain volume, hypertension, diabetes, history of stroke, smoking status, high-density lipoprotein cholesterol, and body mass index). A complementary moderation analysis examined whether the treatment effect of intensive BP control differed by levels of SBP-VIM. The interaction term between treatment assignment and continuous SBP-VIM was tested, and the Johnson-Neyman technique was applied to identify the SBP-VIM range where intensive BP control exerted a significant effect.

The proportion of missing data for baseline covariates ranged from 0% to 1.8%, whereas outcome data were complete (eTable 1). Missing baseline covariates were imputed using multiple imputation (MICE package in R) under a missing-at-random assumption to minimise bias from incomplete data. Model assumptions were assessed by visual inspection of diagnostic plots, including a normal Q-Q plot for normality, a studentized residuals-versus-fitted-values plot for homoscedasticity, a studentized residuals-versus-exposure plot for linearity, and sorted Cook's distances for influential observations (eFigure 2). Statistical significance was defined as a two-sided *p*-value <0.05. All analyses were conducted using R version 4.5.0 (R Foundation for Statistical Computing).

### Data Availability

The deidentified individual participant data underlying the results reported in this article, including relevant clinical and imaging data from the parent trials, are available through the National Heart, Lung, and Blood Institute Biologic Specimen and Data Repository Information Coordinating Center (BioLINCC^19^) to qualified investigators in accordance with BioLINCC data access policies. Derived data generated for the present analysis are available from the corresponding author upon reasonable request for purposes of replicating procedures and results, subject to institutional approval and a signed data access agreement. The original trial protocols and related study documents are available through the parent trial publications and the BioLINCC repository.

## Results

### Cohort Characteristics

**Figure 1:**
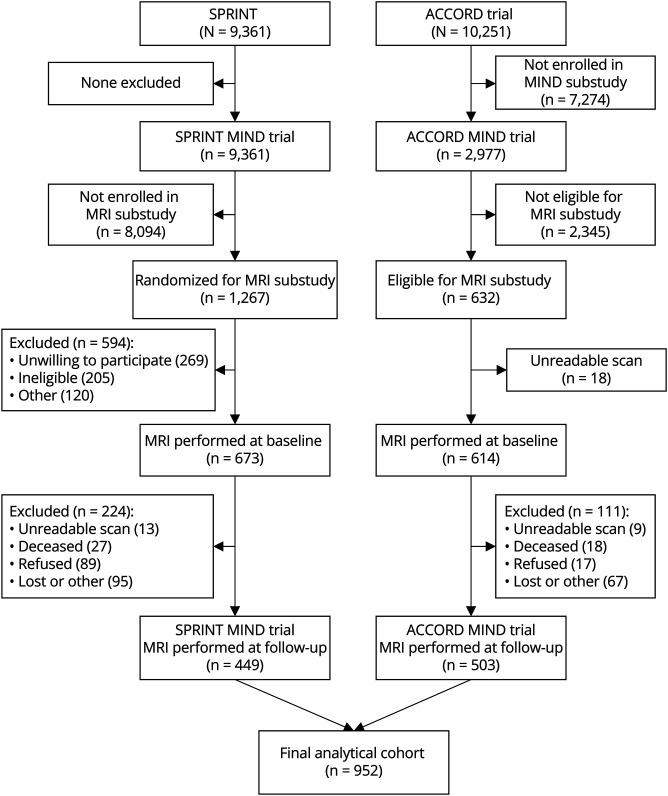
Study Flowchart ACCORD= Action to Control Cardiovascular Risk in Diabetes; SPRINT= Systolic Blood Pressure Intervention Trial; ACCORD MIND= Action to Control Cardiovascular Risk in Diabetes (ACCORD) Memory in Diabetes (MIND); SPRINT MIND= Systolic Blood Pressure Intervention Trial Memory and Cognition in Decreased Hypertension.

A total of 952 participants with complete follow-up MRI were included, comprising 449 from SPRINT MIND trial and 503 from ACCORD MIND trial (Figure 1). Baseline characteristics of MRI substudy participants included in this analysis compared with the parent trial populations are presented in eTable 2, showing only modest differences. Baseline characteristics according to follow-up MRI completion status are shown in eTable 3; participants without follow-up MRI had a less favorable vascular risk profile overall. Baseline characteristics of participants included in this pooled analysis are summarized in Table 1. The mean age was 64.8 years (SD 7.1); 400 (42.0%) were women, and 628 (66.0%) were of White, non-Hispanic ethnicity. Participants in the highest tertile of BP variability were more often female (54.7%) and more frequently Black (23.9%) compared with those in the lowest tertile (31.2% and 19.6%, respectively) and had higher comorbidity burden and baseline SBP, smaller baseline total brain volume, and greater baseline and follow-up AWM volumes.

**Table 1:** Participant Characteristics by Tertiles of Systolic Blood Pressure Variability

Variable	Total N = 952	Tertile1 N = 317	Tertile2 N = 317	Tertile3 N = 318
SBP-VIM, mean (SD)	10.89 (3.80)	7.13 (1.41)	10.36 (0.85)	15.17 (2.78)
Age, y, mean (SD)	64.8 (7.1)	64.5 (6.8)	64.8 (7.2)	65.1 (7.4)
Female sex, No. (%)	400 (42.0)	99 (31.2)	127 (40.1)	174 (54.7)
Ethnicity, No. (%)				
White	628 (66.0)	230 (72.6)	196 (61.8)	202 (63.5)
Black	223 (23.4)	62 (19.6)	85 (26.8)	76 (23.9)
Hispanic	53 (5.6)	14 (4.4)	17 (5.4)	22 (6.9)
Other	48 (5.0)	11 (3.5)	19 (6.0)	18 (5.7)
Comorbid conditions, No. (%)				
Hypertension	816 (85.7)	260 (82.0)	274 (86.4)	282 (88.7)
Diabetes	511 (53.7)	145 (45.7)	187 (59.0)	179 (56.3)
Heart failure	23 (2.4)	4 (1.3)	9 (2.8)	10 (3.1)
Atrial fibrillation	70 (7.4)	18 (5.7)	20 (6.3)	32 (10.1)
Current smoker	108 (11.3)	25 (7.9)	38 (12.0)	45 (14.2)
History of cardiovascular disease	180 (18.9)	48 (15.1)	58 (18.3)	74 (23.3)
History of stroke	17 (1.8)	1 (0.3)	9 (2.8)	7 (2.2)
Body mass index, median (IQR), kg/m^2^	30.5 (27.3–34.6)	30.4 (27.2–33.8)	30.3 (27.4–34.6)	30.9 (27.1–35.2)
Baseline SBP, mean (SD), mm Hg	135.7 (17.3)	133.9 (15.3)	135.4 (16.6)	137.8 (19.4)
Baseline DBP, mean (SD), mm Hg	76.2 (10.9)	76.3 (10.1)	76.0 (10.3)	76.3 (12.3)
Baseline total cholesterol, median (IQR), mg/dL	185.0 (157.0–211.0)	180.0 (158.0–207.0)	187.0 (160.0–212.0)	185.0 (154.0–216.0)
HDL cholesterol, median (IQR), mg/dL	47.0 (39.0–56.0)	46.0 (38.0–55.0)	47.0 (39.0–57.0)	47.0 (39.0–56.0)
Baseline total brain volume, median (IQR), mL	1,019.73 (911.27–1,133.12)	1,068.88 (947.50–1,186.61)	1,013.49 (920.60–1,114.29)	985.46 (875.52–1,104.54)
Baseline AWM volume, median (IQR), mL	1.69 (0.70–4.06)	1.58 (0.70–3.65)	1.60 (0.64–3.76)	1.81 (0.76–4.96)
Baseline CSVD burden, mean (SD)	6.05 (8.42)	4.97 (6.86)	5.85 (7.77)	7.33 (10.15)
Baseline AWM volume, mean (SD), asinh-transformed (mL)^a^	0.81 (0.96)	0.70 (0.87)	0.79 (0.99)	0.94 (1.00)
Follow-up AWM volume, median (IQR), mL	2.58 (1.22–5.44)	2.29 (1.10–4.51)	2.56 (1.04–4.51)	2.95 (1.22–6.72)
Change in AWM volume, median (IQR), mL	0.80 (0.20–2.02)	0.71 (0.19–2.00)	0.70 (0.20–1.90)	0.90 (0.20–2.25)
Annual change in AWM volume, mean (SD), mL/y	0.43 (0.84)	0.33 (068)	0.44 (0.91)	0.52 (0.89)

Abbreviations: AWM = abnormal white matter; CSVD = cerebral small vessel disease; DBP = diastolic blood pressure; HDL = high density lipoprotein; IQR = interquartile range; SBP = systolic blood pressure; VIM = variation independent of mean.

Data are mean (SD), median (IQR), or n (%).

Missing data were minimal (<2% for all variables). Specifically, missing values were present for heart failure (n = 5, 0.5%), atrial fibrillation (n = 17, 1.8%), current smoker (n = 1, 0.1%), body mass index (n = 1, 0.1%), baseline blood pressure (n = 4, 0.4%), total cholesterol (n = 3, 0.3%), high-density lipoprotein cholesterol (n = 3, 0.3%), and baseline total brain volume (n = 4, 0.4%); all other variables had no missing data.

a asinh, inverse hyperbolic sine transformation, f(*x*) = log(*x* + (*x*^2^ + 1)^0.5^).

BP metrics and imaging outcomes in this pooled cohort and by individual trials are summarized in eTable 4. Each participant contributed a median of 12 (IQR 10–14) BP measurements. During follow-up, mean BP was 126.6/69.7 mm Hg. Baseline AWM volume was higher in SPRINT MIND than in ACCORD MIND (3.12 mL vs 0.90 mL), with a corresponding difference in CSVD burden (7.90 vs 4.41). Despite absolute differences, both cohorts showed consistent WML progression. In the pooled analysis, median AWM volume increased from 1.69 mL to 2.58 mL, corresponding to an annualized increase of 0.43 mL per year (SD 0.84), with 0.45 (0.83) mL per year in ACCORD MIND and 0.41 (0.84) mL per year in SPRINT MIND.

### Association Between BPV and AWM Volume Progression

Higher SBP-VIM was significantly associated with greater AWM volume progression (Table 2). Each unit increase in SBP-VIM was associated with a higher asinh-transformed AWM volume change (β = 0.017, 95% CI 0.001 to 0.033; *p* = 0.032). Participants in the highest SBP-VIM tertile had significantly greater AWM volume progression than those in the lowest (β = 0.209, 95% CI 0.061 to 0.357; *p* = 0.006). Similar results were observed using alternative systolic BPV metrics (SD, CV, ARV; eTable 5). In formal heterogeneity analyses, the association between SBP-VIM and AWM progression differed by trial (*p* for interaction 0.013), with significant associations observed in SPRINT-MIND but not in ACCORD-MIND (eTable 6).

**Table 2:** Association Between Systolic Blood Pressure Variability and Change in Abnormal White Matter Volume

Outcomes	Mean (SD)	Unadjusted		Model 1		Model 2		Model 3	
		β (95% CI)	*p* Value	β (95% CI)	*p* Value	β (95% CI)	*p* Value	β (95% CI)	*p* Value
Change in AWM volume, asinh-transformed^a^									
Continuous variable, per unit	0.81 (0.96)	0.020 (0.004, 0.036)	0.013	0.018 (0.002, 0.034)	0.024	0.018 (0.002, 0.034)	0.026	0.017 (0.001, 0.033)	0.032
T1	0.70 (0.87)	1 (Ref)							
T2	0.79 (0.99)	0.096 (−0.053, 0.245)	0.208	0.079 (−0.067, 0.225)	0.289	0.082 (−0.062, 0.227)	0.265	0.044 (−0.101, 0.189)	0.552
T3	0.94 (1.00)	0.244 (0.095, 0.392)	0.001	0.230 (0.082, 0.379)	0.002	0.214 (0.067, 0.361)	0.004	0.209 (0.061, 0.357)	0.006
Change in AWM volume									
Continuous variable, per unit	1.57 (3.06)	0.052 (0.001, 0.103)	0.047	0.047 (−0.005, 0.098)	0.074	0.046 (−0.005, 0.097)	0.079	0.044 (−0.008, 0.096)	0.094
T1	1.19 (2.37)	1 (Ref)							
T2	1.59 (3.37)	0.398 (−0.078, 0.874)	0.101	0.342 (−0.129, 0.813)	0.154	0.351 (−0.116, 0.818)	0.140	0.270 (−0.201, 0.741)	0.260
T3	1.93 (3.31)	0.731 (0.256, 1.207)	0.003	0.696 (0.219, 1.174)	0.004	0.651 (0.176, 1.125)	0.007	0.646 (0.165, 1.128)	0.009
Annual change in AWM volume, asinh-transformed^a^									
Continuous variable, per unit	0.34 (0.49)	0.010 (0.002, 0.018)	0.017	0.009 (0.001, 0.017)	0.027	0.009 (0.001, 0.017)	0.028	0.008 (0.001, 0.017)	0.046
T1	0.27 (0.42)	1 (Ref)							
T2	0.33 (0.51)	0.060 (−0.016, 0.136)	0.121	0.051 (−0.024, 0.126)	0.179	0.099 (−0.029, 0.227)	0.129	0.053 (−0.021, 0.127)	0.162
T3	0.40 (0.53)	0.128 (0.052, 0.204)	0.001	0.123 (0.047, 0.199)	0.002	0.170 (0.041, 0.300)	0.010	0.115 (0.039, 0.190)	0.003
Annual change in AWM volume									
Continuous variable, per unit	0.43 (0.84)	0.014 (−0.001, 0.028)	0.057	0.013 (−0.002, 0.027)	0.081	0.012 (−0.002, 0.026)	0.086	0.011 (−0.003, 0.025)	0.132
T1	0.33 (0.68)	1 (Ref)							
T2	0.44 (0.91)	0.110 (−0.020, 0.240)	0.097	0.096 (−0.033, 0.225)	0.143	0.099 (−0.029, 0.227)	0.129	0.065 (−0.064, 0.193)	0.323
T3	0.52 (0.89)	0.190 (0.061, 0.320)	0.004	0.184 (0.053, 0.314)	0.006	0.170 (0.041, 0.300)	0.010	0.160 (0.028, 0.291)	0.017

Abbreviations: AWM = abnormal white matter; SBP = systolic blood pressure; VIM = variation independent of mean.

Results are shown as β (95% CI).

Model 1: age, sex, ethnicity.

Model 2: age, sex, ethnicity, mean SBP during the follow-up period.

Model 3: age, sex, ethnicity, mean SBP during the follow-up period, total brain volume, intensive blood pressure control, hypertension, diabetes, history of stroke, smoking status, high-density lipoprotein cholesterol, and body mass index.

a asinh, inverse hyperbolic sine transformation, f(*x*) = log(*x* + (*x*^2^ + 1)^0.5^).

Consistent associations were observed when AWM volume was analyzed in absolute or annualized terms. Higher SBP-VIM was independently associated with faster annualized asinh-transformed AWM volume progression (β = 0.008, 95% CI 0.001 to 0.017; *p* = 0.046). Participants in the highest SBP-VIM tertile showed both greater absolute AWM volume increase (β = 0.646, 95% CI 0.165 to 1.128; *p* = 0.009) and faster annual asinh-transformed lesion progression (β = 0.115, 95% CI 0.039 to 0.190; *p* = 0.003) and untransformed lesion progression (β = 0.160, 95% CI 0.028 to 0.291; *p* = 0.017) after multivariable adjustment (Table 2). Similar results were observed using alternative systolic BPV metrics, including SD, CV, and ARV (eTables 7–9). In clinically interpretable units, participants in the highest tertile of systolic BPV had 0.646 mL greater cumulative AWM progression and 0.160 mL/y faster annualized progression than those in the lowest tertile; the latter corresponds to approximately 37% of the average annual AWM progression observed in the cohort.

**Figure 2:**
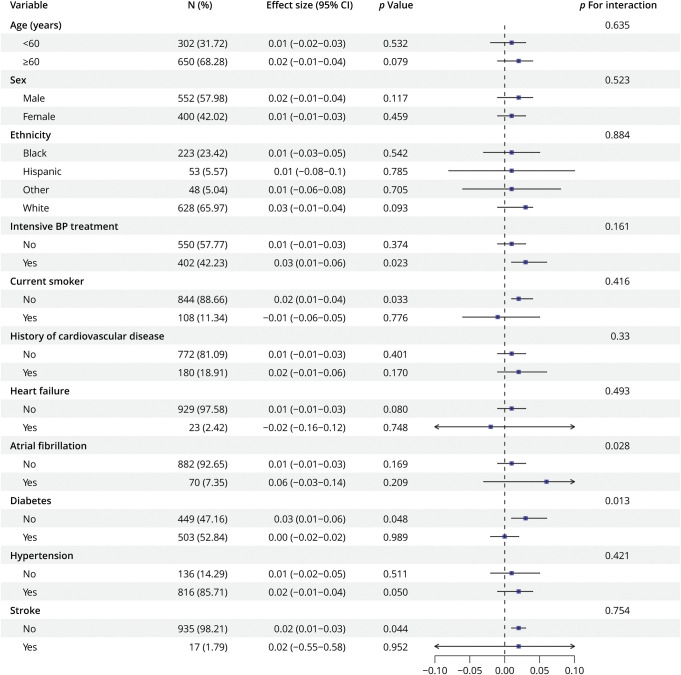
Subgroup Analyses of the Association Between SBP-VIM and Change in AWM Volume* Forest plots display fully adjusted β coefficients (95% confidence intervals) for the association between SBP-VIM and AWM volume change within prespecified clinical subgroups. *p* values represent the main effect within subgroups, and *p* for interaction corresponds to the multiplicative term in multivariable models. AWM = abnormal white matter; BP = blood pressure; SBP = systolic blood pressure; VIM = variation independent of mean. *Change in AWM volume was asinh-transformed.

The association between higher SBP-VIM and greater AWM volume progression was largely consistent across clinical subgroups (Figure 2). No significant interactions were observed for age, sex, ethnicity, intensive BP treatment allocation, or most vascular risk factors (all *p* > 0.05). However, modest effect heterogeneity was observed by diabetes status (*p* = 0.013) and atrial fibrillation (*p* = 0.028), with stronger associations among participants without diabetes or atrial fibrillation.

Restricted cubic spline analysis further characterized the association between systolic BPV and AWM volume progression (eFigure 3). The overall relationship between SBP-VIM and AWM volume progression was statistically significant (*p* for overall = 0.010) and showed modest evidence of nonlinearity (*p* for nonlinearity = 0.034). Within the mid-range of SBP variability, higher SBP-VIM was associated with progressively greater predicted AWM volume change, consistent with the dose-response pattern observed in categorical analyses. At the extremes of SBP-VIM, the curve flattened and slightly oscillated, likely reflecting wider confidence intervals because of smaller numbers of participants. Among other BPV indices, SBP-CV and SBP-ARV showed consistent but modest positive associations with AWM volume progression (*p* = 0.024 and 0.021, respectively), whereas SBP-SD showed no significant association (*p* = 0.111).

### Association Between Intensive BP Control and AWM Volume Progression

Compared with standard treatment, intensive BP control was associated with slower AWM volume progression (Table 3). In unadjusted analysis, participants in the intensive group had smaller increases in asinh-transformed AWM volume (0.7 ± 1.0 mL vs 0.9 ± 0.9 mL; β = −0.247, 95% CI −0.370 to −0.125; *p* < 0.001). This association remained robust after multivariable adjustment (β = −0.270, 95% CI −0.393 to −0.146; *p* < 0.001). Results were consistent for both absolute and annualized change in AWM.

**Table 3:** Association Between Intensive Blood Pressure Control and Change in Abnormal White Matter Volume

Outcomes	Standard SBP control (N = 550)	Intensive SBP control (N = 402)	Unadjusted		Model 1		Model 2	
			β (95% CI)	*p* Value	β (95% CI)	*p* Value	β (95% CI)	*p* Value
Change in AWM volume, asinh-transformed (mL)^a^	0.9 ± 0.9	0.7 ± 1.0	−0.247 (−0.370, −0.125)	<0.001	−0.298 (−0.419, −0.178)	<0.001	−0.270 (−0.393, −0.146)	<0.001
Change in AWM volume, mL	1.8 ± 3.2	1.3 ± 2.8	−0.486 (−0.880, −0.093)	0.016	−0.617 (−1.008, −0.227)	0.002	−0.613 (−1.014, −0.212)	0.003
Change in AWM volume/year, mL/y	0.5 ± 0.9	0.3 ± 0.7	−0.151 (−0.258, −0.043)	0.006	−0.182 (−0.288, −0.075)	<0.001	−0.164 (−0.274, −0.055)	0.003
Change in AWM volume/year, asinh-transformed (mL/y)^a^	0.4 ± 0.5	0.3 ± 0.5	−0.109 (−0.172, −0.047)	<0.001	−0.131 (−0.193, −0.069)	<0.001	−0.116 (−0.180, −0.053)	<0.001

Abbreviations: AWM = abnormal white matter; SBP = systolic blood pressure.

Results are shown as β (95% CI).

Model 1: age, sex, ethnicity.

Model 2: age, sex, ethnicity, total brain volume, hypertension, diabetes, history of stroke, smoking status, high-density lipoprotein cholesterol, and body mass index.

a asinh, inverse hyperbolic sine transformation, f(*x*) = log(*x* + (*x*^2^ + 1)^0.5^).

### Mediation and Moderation by BPV

**Figure 3:**
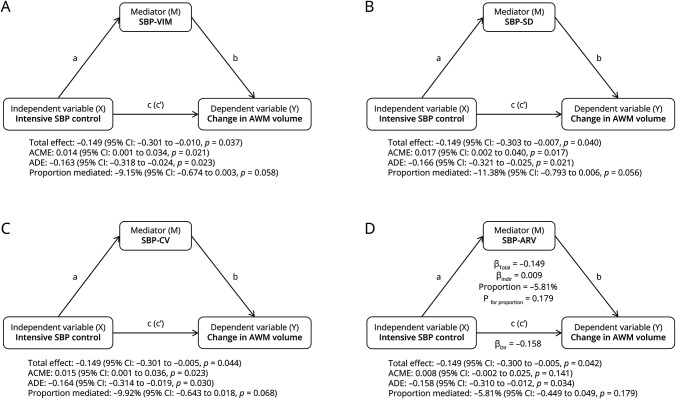
Mediation Analyses of the Association Between Intensive BP Control and AWM Progression Through Systolic BPV Four parallel mediation models evaluated the association between intensive systolic blood pressure (BP) control (X) and change in abnormal white matter (AWM) volume (Y) through different systolic BP variability (BPV) parameters (M): (A) SBP-VIM, (B) SBP-SD, (C) SBP-CV, and (D) SBP-ARV. Intensive BP control showed a consistent direct protective effect (negative ADE) on AWM progression across all models. Significant indirect effects (ACME) were observed for SBP-VIM, SBP-SD, and SBP-CV, indicating suppression, whereby these BPV parameters partially offset the magnitude of the direct effect. No significant mediation was identified for SBP-ARV. ACME = average causal mediation effect; ADE = average direct effect; ARV = average real variability; BP = blood pressure; CV = coefficient of variation; SBP = systolic blood pressure; VIM = variation independent of mean. Change in AWM volume was asinh-transformed in all models.

In mediation analysis, higher SBP-VIM partially mediated the association between intensive BP control and reduced AWM volume progression (ACME = 0.014, 95% CI 0.001–0.034; *p* = 0.021), accounting for 9.15% of the total effect (*p* for proportion = 0.058). The direct effect of intensive BP control remained significant (ADE = −0.163, 95% CI −0.318 to −0.024; *p* = 0.023) (Figure 3), indicating that both BP lowering and variability reduction contributed to the attenuation of white matter injury. Similar results were observed when SBP variability was quantified using SD, CV, or ARV (Figure 3).

In moderation analysis, the unadjusted model revealed a significant interaction between intensive BP control and continuous SBP-VIM (*p* for interaction = 0.036) (eFigure 4). The Johnson-Neyman interval indicated a threshold of 13.95 mm Hg, below which intensive BP control was associated with significantly less AWM volume progression (*p* < 0.05). After full adjustment, the interaction effect was attenuated (*p* for interaction = 0.161).

## Discussion

In this pooled analysis of 2 large, well-characterized randomized controlled trials, we found that among patients with high cardiovascular risk, higher visit-to-visit systolic BPV was independently associated with greater AWM volume progression, irrespective of mean BP levels. Intensive BP control slowed AWM volume progression, and this protective effect was partly mediated by reduced systolic BPV. These findings highlight BPV as an important determinant of cerebral microvascular injury and may provide mechanistic insight into the pathways through which intensive BP control preserves cerebral white matter integrity.

The annual WML progression observed in this pooled analysis is comparable to that reported in a previous meta-analysis, which found progression rates of 0.46 cc/y in community cohorts.^24^ In addition, findings of this study are also consistent with previous observational studies that determined the association between higher BPV and greater WML progression^8,17,18^ and extend this evidence by leveraging individual-level data from 2 rigorously conducted randomized trials with standardized BP monitoring, centrally adjudicated MRI outcomes, and multiple measurements of change in AWM volume (including both absolute and annualized, and asinh-transformed and untransformed). In addition, consistent with the individual SPRINT MIND and ACCORD MIND results,^11,12^ this pooled analysis reinforces the neuroprotective effects of intensive BP control on preserving white matter integrity. By quantifying AWM, a composite measure encompassing both overt lesions and subtler tissue abnormalities and enhancing detection of microvascular injury related to BP dynamics, it provides a more sensitive and integrative marker of cerebral WML.^22,25^

Consistent with previous studies,^13^ the present analysis confirmed that intensive BP lowering slowed the progression of WML and, through mediation analysis, provides novel mechanistic insights into the neuroprotective pathways underlying this effect. The negative mediation coefficient indicates that intensive BP control simultaneously both reduced BPV and slowed WML progression. These results highlight that beyond achieving lower mean BP, maintaining hemodynamic stability may play a complementary role in preserving cerebral microstructure. Moreover, this finding may also help explain a common clinical phenomenon in which some patients show rapid WML progression despite apparently well-controlled mean BP, possibly reflecting, at least in part, the contribution of residual BP fluctuations. These results underscore that both the magnitude and stability of BP reduction are important and that strategies optimizing both may enhance cerebrovascular protection.

In the context of previous evidence linking BPV with cerebrovascular disease and cognitive decline,^26^ our findings further highlight BPV as a potentially modifiable target for cerebral protection. Although current hypertension management primarily focuses on achieving lower mean BP, maintaining BP stability over time may offer additional neuroprotective benefits. Emerging evidence suggests that calcium channel blockers may attenuate BP fluctuations, whereas β-blockers may increase them, implying class-specific effects on WML progression, although definitive clinical evidence of their neuroprotective effects remains limited.^27^ Future prospective and interventional studies are needed to determine whether stabilizing BPV can slow white matter degeneration and mitigate cognitive decline. In addition, incorporating BPV metrics into clinical monitoring may help identify individuals at higher risk for CSVD and guide a more individualized, stability-oriented approach to BP management.

Subgroup analyses revealed significant effect modification by diabetes status, with stronger associations between BPV and WML progression in participants without diabetes. Although the calculation of BPV is different, this is consistent with post hoc findings from the ACCORD MIND trial showing no significant association between BPV and AWM in type 2 diabetes,^12^ and further supports diabetes as a key modifier of the cerebrovascular effects of BPV. One possible explanation is that long-standing diabetes-related microvascular pathology, including endothelial dysfunction, capillary rarefaction, and impaired cerebral autoregulation, may overshadow the incremental effect of BPV on white matter integrity.^28^ In contrast, in nondiabetic hypertensive individuals, fluctuations in BP might more directly translate into intermittent cerebral hypoperfusion and white matter injury. These results may indicate that BPV may represent a modifiable therapeutic target primarily in nondiabetic individuals, whereas in diabetes, white matter injury appears to be driven by distinct microvascular mechanisms. However, it should be noted that differences in antihypertensive treatment regimens or other unmeasured factors may also contribute to the observed heterogeneity between diabetic and nondiabetic participants.

The rationale for pooling SPRINT-MIND and ACCORD-MIND is supported by several key similarities in design and measurement. Both were randomized, SBP target–driven trials comparing intensive vs standard BP control, with the same SBP target. In both trials, BP was measured using protocol-specified clinic assessments at approximately 2- to 4-month intervals during follow-up, enabling harmonized derivation of visit-to-visit systolic BPV. MRI outcomes were derived using automated, atlas-based segmentation pipelines in both cohorts, and AWM metrics were available in both trials. Follow-up durations were comparable and sufficiently long to capture lesion progression. Finally, both cohorts comprised adults at elevated cardiovascular risk; although diabetes status differed by design, this broadened the clinical spectrum represented in the pooled analysis and enabled assessment of potential effect modification.

This study also has limitations. First, both SPRINT and ACCORD enrolled participants at high cardiovascular risk and few of them experienced stroke, which may limit generalizability to lower-risk populations or patients with stroke. Second, both trials were SBP target–driven and did not mandate specific antihypertensive regimens; therefore, residual confounding related to antihypertensive regimen cannot be fully excluded. Third, although we analyzed AWM progression rather than cross-sectional lesion burden, reverse causation cannot be fully excluded, as underlying CSVD or preexisting white matter injury may itself contribute to greater BPV. This concern is particularly relevant given the approximately 4-year follow-up and warrants cautious causal interpretation of the observed associations. Fourth, MRI-derived AWM measures were generated using different postprocessing pipelines in both trials; therefore, residual methodological heterogeneity related to image acquisition and processing cannot be fully excluded. Finally, as a post-hoc analysis within randomized trials, residual confounding cannot be entirely excluded despite extensive covariate adjustment; moreover, because MRI substudy participants differed modestly from the parent trial populations and those without follow-up MRI had a less favorable vascular risk profile and higher BPV, some selection and attrition bias may remain despite multivariable adjustment.

In patients with high cardiovascular risk, higher visit-to-visit systolic BPV was independently associated with faster WML progression, irrespective of mean BP levels. Intensive BP control mitigated white matter injury, and this protective effect was partly mediated by reduced systolic BPV. Future studies are warranted to determine whether direct interventions targeting BPV can further protect cerebral white matter and elucidate the additional mechanisms underlying the benefits of intensive BP control on microvascular integrity.

## Glossary

ACCORD: Action to Control Cardiovascular Risk in Diabetes
ACME: average causal mediation effect
ADE: average direct effect
ARV: average real variability
AWM: abnormal white matter
BP: blood pressure
BPV: blood pressure variability
CSVD: cerebral small vessel disease
CV: coefficient of variation
IQR: interquartile range
VIM: variation independent of mean
WML: white matter lesions
